# Cognitive Effects of Aromatase and Possible Role in Memory Disorders

**DOI:** 10.3389/fendo.2018.00610

**Published:** 2018-10-17

**Authors:** Cheryl S. Rosenfeld, Dusti A. Shay, Victoria J. Vieira-Potter

**Affiliations:** ^1^Bond Life Sciences Center, University of Missouri, Columbia, MO, United States; ^2^Thompson Center for Autism and Neurobehavioral Disorders, University of Missouri, Columbia, MO, United States; ^3^Biomedical Sciences, University of Missouri, Columbia, MO, United States; ^4^Nutrition and Exercise Physiology, University of Missouri, Columbia, MO, United States

**Keywords:** E2, brain, rodent models, breast cancer, Letrozole, Fadrozole, Alzheimer's Disease

## Abstract

Diverse cognitive functions in many vertebrate species are influenced by local conversion of androgens to 17β-estradiol (E2) by aromatase. This enzyme is highly expressed in various brain regions across species, with some inter-species variation in terms of regional brain expression. Since women with breast cancer and men and women with other disorders are often treated with aromatase inhibitors (AI), these populations might be especially vulnerable to cognitive deficits due to low neuroE2 synthesis, i.e., synthesis of E2 directly within the brain. Animal models have been useful in deciphering aromatase effects on cognitive functions. Consequences of AI administration at various life cycle stages have been assessed on auditory, song processing, and spatial memory in birds and various aspects of cognition in rodent models. Additionally, cognitive deficits have been described in aromatase knockout (ArKO) mice that systemically lack this gene throughout their lifespan. This review will consider evidence to date that AI treatment in male and female rodent models, birds, and humans results in cognitive impairments. How brain aromatase regulates cognitive function throughout the lifespan, and gaps in current knowledge will be considered, along with future directions to better define how aromatase might guide learning and memory from early development through the geriatric period. Better understanding the importance of E2 synthesis on neurobehavioral responses at various ages will likely aid in the discovery of therapeutic strategies to prevent potential cognitive deficits, including Alzheimer's Disease, in individuals treated with AI or those possessing *CYP19* gene polymorphisms, as well as cognitive effects of normal aging that may be related to changes in brain aromatase activity.

## Introduction

Aromatase is the enzyme responsible for all endogenous 17β-estradiol (E2) production in both males and females. Importantly, is it expressed in the brain as well as peripheral organs (i.e., gonads and adipose tissue). E2 has a diverse array of effects on brain development and function throughout the lifespan, including promoting development and maintenance of various cognitive abilities (1, 2); thus, it is not surprising that compounds designed to suppress aromatase can exert deleterious cognitive effects. Such cognitive effects of aromatase inhibition might vary according to species (e.g., learning and memory of vocalizations in songbirds) but there also are some consistencies across species that this review will highlight. This review will consider various cognitive effects in avian and rodent models, as well as the epidemiological evidence to date in humans, especially women, who received these chemicals and showed later cognitive impairments.

**Figure 1:**
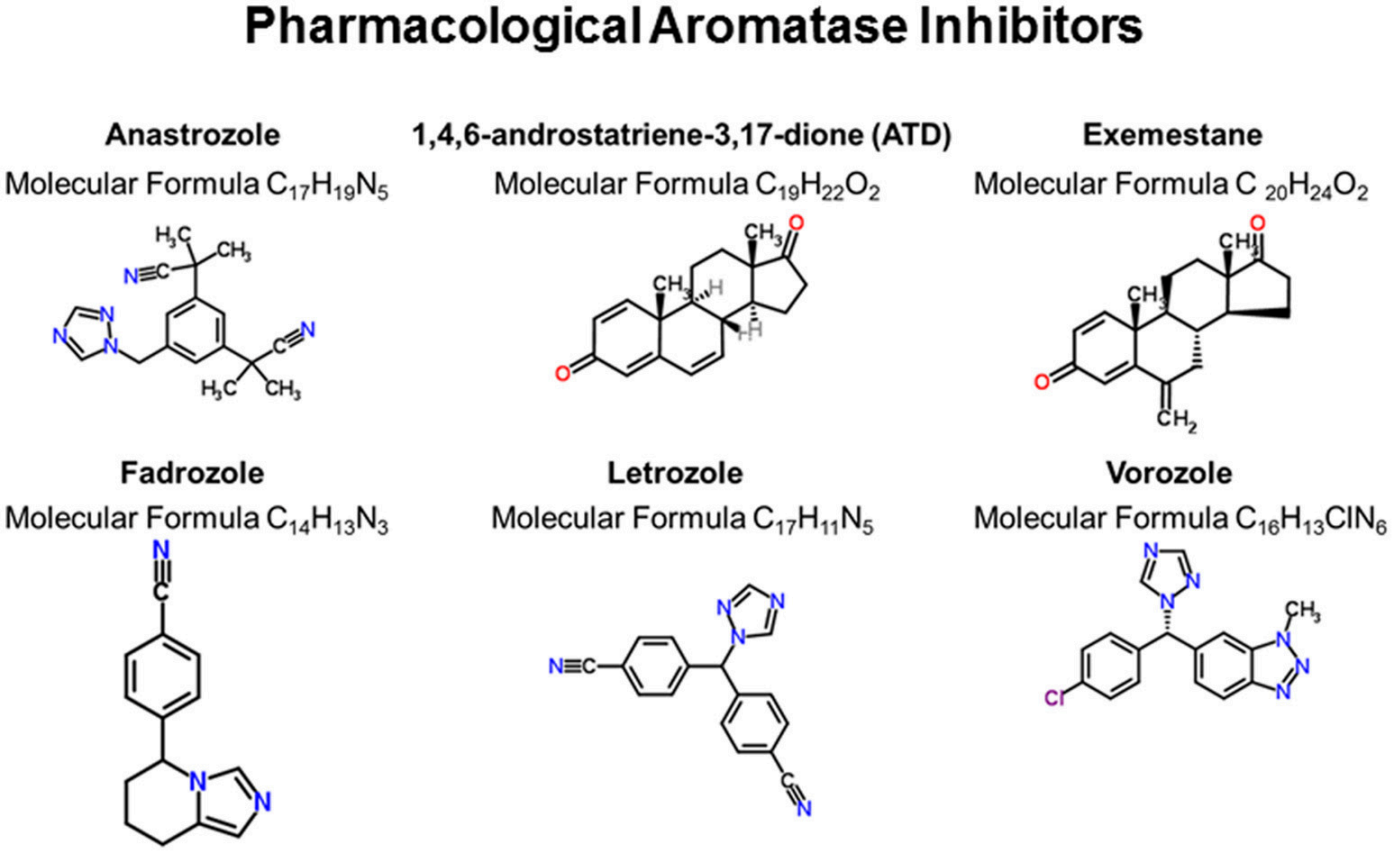
Pharmacological inhibition of aromatase. These six compounds represent the primary ones used to inhibit aromatase activity in animals and humans. Chemical structures were generated from the ChemSpider webpage (http://www.chemspider.com/).

Women with breast cancer and individuals of both sexes with various other disorders (e.g., short stature in boys, infertility in men and women, endometriosis, leiomyomatosis, and Klinefelter syndrome) are increasingly being administered aromatase inhibitors (AI) to suppress conversion of androgens to estrogens (3–9). While such pharmacological therapies have been useful adjuvants in treating these disorders, there is mounting concern that systemic suppression of aromatase can lead to adverse health effects, especially cognitive dysfunction and other neurological symptoms (10–18). AI compounds that have been used in women and tested in animal models are Anastrazole (An), 1,4,6-androstatriene-3,17-dione (ATD), Exemestane, Fadrozole (FAD), Letrozole (Letro), and Vorozole. The chemical structures and molecular information are shown in Figure 1.

E2is considered essential in preserving normal brain function throughout life and may also play a role in recovery from brain injury or stress (2, 19–22). Early development brain programming is considered the organizational period and is characterized by rising concentrations of androgens, and aromatase conversion of androgens to estrogens within various brain regions (23–26), as detailed below. Timing is critical when considering how E2 affects brain development. As an example, most sex-dependent behaviors generally require a later resurgence of such steroid hormones (“activational period”) to stimulate sex-specific neuronal pathways. AIs can disrupt this normal organizational-activational programming of the brain (27, 28). Unfortunately, most of the animal and human studies investigating the effects of AI on various outcomes related to cognitive processes are based on administration of AIs to adults. Thus, a topic that requires more study is how AI during early development, and even *in utero* (e.g., exposure during pregnancy of compounds that may advesely affect aromatase) may affect cognition during the lifespan.

In the case of rodents, studies have examined the effects of life-long aromatase deficiency using genetically modified mice that lack the *Cyp19* gene, which encodes for the aromatase enzyme. Such mice lack the gene from the time of conception and throughout their lifespan (29–32). A limitation of those studies, as well as some human studies that have assessed genetic polymorphisms in *Cyp19*, is that it is often difficult to distinguish those effects that are directly due to aromatase inhibition within the brain vs. other organs, including the gonads, which might indirectly affect neural responses. Aromatase knock-out (ArKO) mice lack *Cyp19* in all organs, including the brain; thus, cognitive disruptions observed in this model might be due to *Cyp19* ablation within the brain and/or other organs. To address this issue, some rodent studies (discussed later) have directly infused aromatase into specific brain regions of gonadectomized animals. We will also discuss methods to disentangle the direct effects of aromatase inhibition in the brain vs. indirect effects due to suppression of this enzyme within other target organs.

In relation to the human studies, we also explore potential strategies, namely physical exercise, that might be useful in combating the memory and other neurological effects induced by AI. We consider the human epidemiological and animal model studies linking aromatase suppression or ablation with Alzheimer's disease (AD). Finally, we consider whether certain cognitive functions are influenced by aromatase suppression across taxa, discuss potential limitations of the current studies, and suggest future directions that are necessary to address the outstanding questions in this area that are increasingly becoming important with greater usage of such pharmacological agents. The focus of this review is the role of aromatase in regulating cognitive behaviors. Indeed, abundant literature implicates E2 in regulating various cognitive behaviors, especially in females (1, 2, 19, 33–35). It is, however, beyond the scope of the current work to delve into the various mechanisms by which estrogens might affect learning and memory, including through membrane and/or nuclear estrogen receptors (GPER, ESR1, and ESR2).

## Aromatase expression in the brain: *a brief history and overview*

In 1971, Naftolin et al., first established the presence of aromatase in human fetal hypothalamus and within a few years developed the aromatization hypothesis, which describes the conversion of testosterone (T) to E2 via aromatase and its role in sexual differentiation of the brain (36). The second aromatization hypothesis that was put forward by two independent groups suggested that this enzyme was essential in the induction of masculine sexual behavior governed by E2 (37, 38). This enzyme has now been discovered in several other brain regions. Under physiological conditions, aromatase is constitutively expressed in the neurons of reptiles, birds, and mammals (35). In mammals, CYP19 expression/aromatase activity has been detected in the hypothalamus, medial preoptic area, bed nucleus of stria terminalis (BST), MeA, prefrontal cortex, hippocampus, and cerebellum (35, 39–46). Various brain regions of birds tend to express higher amounts of aromatase than in mammals, and thus avian models have greatly expanded our knowledge of how this enzyme regulates cognitive and other behavioral processes (35). Interestingly, studies examining the co-localization of aromatase mRNA and estrogen receptors in avian and mammalian species have demonstrated correlations between aromatase and estrogen receptor concentration, which differ based on species and brain region (45, 47, 48).

## Species and sex differences in aromatase expression and activity

Male rats and mice tend to have higher levels of aromatase expression to convert androgens to estrogens to induce masculine sexual behavior (46, 49–51). However, the levels of aromatase expression and activity differ greatly across brain region and between sexes, in addition to differences in sensitivity to sex-hormone status across the lifespan. This sex difference is most important in brain areas responsible for guiding sexual differentiation and regulating socio-sexual behaviors later in life, such as the hypothalamus and amygdala.

The affinity of brain aromatase to androgen substrates is identical in male and female quail brain (52). In avian species, varying patterns in brain aromatase expression are observed, which, again, differ by species and brain region examined. Male and female Japanese quail (*Coturnix japonica*) express similar *Cyp19* mRNA levels in the medial preoptic area (MPOA) and mediobasal hypothalamus (MBH), but females have reduced protein expression in the MPOA region (53, 54). In zebra finches (*Taeniopygia guttata*), females exhibit an increased number of aromatase-positive soma than males in the hypothalamus-preoptic area (HPOA) and caudomedial nidopallium (NCM) but fewer immuno-positive cell processes and synapses (43, 55). Similar results for aromatase activity have been reported in the synaptic terminals of female zebra finches (56). Relative to body size, avian species demonstrate substantially greater aromatase activity across most brain regions when compared to mammals (35, 52, 57, 58). Studies examining *CYP19* mRNA expression patterns in humans have generated inconsistent results. Whereas Kadioglu et al. (59) and Sasano et al. (60) found no differences between male and female *CYP19* mRNA expression across several brain regions examined, including pons, thalamus, hypothalamus, hippocampus, and amygdala.

In male rodents (e.g., *Rattus norvegicus*), ring doves (*Streptopelia risoria*), and Japanese quail, T and its metabolites regulate the long-term expression of aromatase in various brain regions, such as the amygdala, BNST, POA, and ventromedial nucleus of the hypothalamus, and medial preoptic nucleus (POM) (61–64). Both E2 and dihydrotestosterone (DHT) regulate *Cyp19* mRNA levels only in XX mice independently of gonadal sex (46). On the other hand, in female rodents and birds, both T and E2 appear to regulate neural *Cyp19* mRNA, protein, and activity levels (62, 65–72). In terms of non-genomic actions, cellular alterations in potassium, glutamate, and calcium-dependent phosphorylation pathways have been shown to induce short-term changes in brain aromatase activity, as primarily shown in birds and rats (35, 73–82).

## Cognition assessment techniques commonly used in animal studies

To test cognitive ability in rodent models, most tests, such as the Barnes, Morris Water Maze (MWM), Y and radial arm mazes, measure spatial learning and memory. In such tests, individuals are repeatedly tested over a multi-day period, and their ability to locate an escape hole or maze arm that remains the same over the test is measured with the presumption that their latency to locate the correct hole or arm of the maze should decline with learning (83–85). Another common test to measure cognition in rodents is social recognition or their ability to differentiate novel animals of the same sex or different sex (86). The studies detailed below in ArKO mice, or rodents administered an AI, provide examples of results from such cognitive tests. Songbirds have been useful in examining how AI can affect learning as it relates to song vocalization, internal processing, and response rate, as these are key cognitive functions in this species. Other avian studies have measured the effects of AI on spatial learning and memory but with methods differing from those used in rodent studies. Such spatial studies are critical in bird species that cache their food over the winter period.

## Rodent models of aromatase ablation/suppression

To examine the role of aromatase in various organs, including the brain, aromatase knockout (ArKO) mice have been created by two separate groups (29–32). Both ArKO mouse models have targeted deletion and insertion of a neomycin-resistance gene within exon 9 with a few more nucleotides replaced in the first model (29) compared to the second model generated (30). Even so, no studies have compared the two ArKO models in the same study to determine if there are subtle endophenotypic differences, and all of the studies detailed below are based on the first model. Both male and female ArKO mice show cognitive deficits when tested in a Y-maze to assess short-term spatial reference memory (87). Gonadectomy suppressed spatial responses of wild-type (WT) mice compared to that of intact ArKO counterparts, but this surgery did not exacerbate any impairments in ArKO mice relative to intact ArKO individuals. On the other hand, no changes in spatial learning ability were identified in adult ArKO female mice tested in the Morris water maze (MWM) even though increased expression of N-methyl-D-aspartase receptor subunit (*Nr2b*) was present in the hippocampus of female ArKO compared to WT mice (88). Based on these two studies, it is difficult to draw definitive conclusions as to whether ArKO mice demonstrate deficits in spatial learning and memory, although the latter study supports the notion that aromatase enzyme is important in initial male sexual differentiation as aromatase removal had no effect in females. The Y-maze assesses short-term memory, whereas, the MWM measures spatial memory over a multi-day trial period. However, it could be argued that the MWM is not considered an ethologically relevant test for terrestrial rodents (89). A better approach might be to examine ArKO mice in the dry-land Barnes maze. It is also clear from other rodent studies that sex differences can exist in spatial learning and memory (90–92). Therefore, in future studies, both male and female ArKO mice should be tested in this maze and compared to their WT counterparts.

Potential socio-cognitive disruptions have also been examined in ArKO mice. Social recognition was diminished in ArKO male mice with associated reduction in arginine vasopressin (AVP, which as it is similar in structure to oxytocin can play a role in social behaviors and recognition)-immunoreactivity (IR) in several brain regions (93). Adult treatment with estradiol benzoate and dihydrotestosterone propionate (DHTP) restored this cognitive ability in gonadetomized ArKO males, which then showed rescue of their normal responses to female-oriented ultrasonic vocalizations, and were able to recognize unfamiliar females. Estradiol benzoate and DHTP treatment increased AVP-IR in the lateral septum of these males.

Olfactory discrimination ability is another important component in social cognition. WT males and ArKO males and females learned more rapidly to distinguish between urinary odors collected from gonadally intact males and estrous females compared to WT females (94). Of those groups, ArKO females were the quickest to acquire the ability to recognize volatile urinary odors from ovariectomized (OVX) and E2/progesterone vs. E2 alone-treated females. However, another study showed that while both ArKO and WT female mice can discriminate between odors of two different males, ArKO females failed to learn familiar male odors (95). This deficit might be due to reduced neurogenesis in the accessory olfactory bulb of ArKO females (95), suggesting that aromatase conversion of T to E2 is required for neuronal cell proliferation of this brain region in females. Taken together, the findings suggest that ArKO males may have decreased reproductive success because of impaired ability to distinguish individual female smells. However, ability to discern sex-differences in urinary odors appears to be enhanced in ArKO males and females with the latter possibility exhibiting masculinization of this trait. While females can discern male odors, they may exhibit memory impairments regarding familiar male odors, which could potentially also compromise their reproductive success. Moreover, synapse density is reduced in the hippocampus of female but not male ArKO mice (96). Thus, such enhanced responses in ArKO female might be due to ablation of central aromatase activity, i.e., E2 produced within the ovary, or due to masculinization in other brain regions. While such ArKO mice studies are helpful in discerning the role of brain aromatase in the case of gonadectomized individuals, these mice lack this enzyme throughout their lifespan, and thus, the effects of E2 deficiency at different critical time-periods, including at development vs. adulthood, cannot be distinguished. For such reasons, rodent models that use pharmacological inhibition of aromatase are the ideal choice to address such issues, at least until temporal and brain specific *Cyp19* mice are generated. One such study that the administered the AI, ATD, to neonatal rats provides further support that aromatase is essential in male sexual differentiation of select neurons, such as those within the central portion of the vomeronasal projection circuit, and guiding later response to pheromonal cues (97). Other rodent studies testing various AI are detailed below.

In one study investigating working memory, adult female rats were OVX and then administered vehicle alone, testosterone propionate (TP) alone, Letro only or the combination of TP + Letro (98). Intact females demonstrated the best working memory in the object recognition test compared to all other groups with no differences among any of the OVX groups. The collective findings suggest that the observed cognitive responses are due to AI treatment primarily affecting ovarian rather than brain aromatase activity. Findings from other studies with intact male rats suggest that AI treatment might actually improve working memory. In one study, Sprague-Dawley adult male rats were orally administered Letro or vehicle (99). Unexpectedly, those treated with Letro committed fewer errors in a cross-arms maze. The investigators concluded that Letro may improve egocentric working memory, which is where memories are encoded in how objects in space relate to position of oneself as opposed to allocentric- encoding of object information based on relation to other objects, by raising T levels. Adult male albino Wistar rats were administered the AI, An, directly into the CA1 region of the hippocampus (100). Other males were treated with testosterone enanthate (TE), E2 valerate (EV), or dimethylsulfoxide (DMSO, vehicle) alone. Those treated with the highest doses of TE or EV showed an increase in escape latency and traveled greater distance to find the invisible platform, whereas the AI, An, led to dose dependent decreases in escape latency and travel distance to find the hidden platform. These findings further indicate that AI in intact males might actually improve cognitive abilities, possibly by suppressing E2 synthesis.

Six- to eight-week old Swiss albino female mice were treated for 10–15 days with three different AIs that have different structures but similar potencies: An, Letro, or exemestane, and controls received just the vehicle solution. Both An and Letro but not exemestane suppressed learning and memory in the MWM. The behavioral changes correlated with expression levels for dickkopf1 (*Dkk1*, an inhibitor of the WNT signaling pathway) and sclerostin (*Sost*, which is known to exert anti-anabolic effects on bone formation but uncertain CNS effects) in the hippocampus (101). Select studies have found strain or species-specific effects of AI, which could also depend upon dose of drug tested. While Letro treatment for 3 weeks induced anxiety-like behaviors in OVX C57Bl6/6J mice, this treatment improved spatial learning and memory in the MWM (102). Young adult OVX Fischer-344 virgin female rats treated with An (showed no cognitive disturbances when tested in the water radial-arm maze (WRAM), delay match to sample (DMS) three choice task, MWM, and visible platform maze, tests designed to measure spatial and non-spatial working memory (103). Collectively, these studies do not provide a clear picture of how AI might affect these cognitive responses. The disparate responses across these studies are likely due to variation in the strain of mouse or rat tested, AI administered, dose and duration of treatment, and specific cognitive test. While each of these tests tend to measure hippocampal-memory, how the tests are conducted, age of the animals tested, and order of the behavioral tests are also important variables to consider.

Administration of Letro to adult C57Bl/6J male mice downregulated steroid receptor coactivator-1 (*Src1*, which mediates transcriptional activity of nuclear receptors) in brain regions related to learning and memory integration and other behaviors, medial septa, hippocampus, medial habenular nucleus, arcuate hypothalamic nucleus, and superior colliculus (11). Circulating levels of E2 and gene expression of aromatase (i.e., *Ar)*, as well as E2 receptors alpha and beta (i.e., *Esr1 and Esr2*, respectively) were also decreased in the hippocampus of Letro-treated mice. Similarly, Letro treatment for 1 week to adult female Wistar rats suppressed *Src1* and postsynaptic density protein 95 (*Psd95*) in the hippocampus (104). RNA interference with *Src1* in *in vitro* culture of primary hippocampal neurons has been shown to inhibit *Psd95* gene and protein expression, suggesting that *Src1* modulates the effects E2 synthesis of hippocampal synaptic plasticity via the synaptic protein PSD95.

To distinguish between the role of neural vs. the role of gonadal aromatase on cognitive responses, AIs have been administered directly into the brain of gonadectomized rodent models. Bilateral Letro infusion into the dorsal hippocampal region of OVX female rats immediately after training impairs hippocampal memory consolidation for object recognition and placement (105). This treatment also suppressed the surge in dorsal hippocampal E2 synthesis, generally observed 30 min after object training. Intracerebroventricular infusion of Letro into the brain of OVX rats did not affect learning responses in control individuals, but this treatment prevented exogenous estradiol treatment from enhancing hippocampal-dependent spatial memory acquisition in a radial-maze task (106). The combined findings from the two studies suggest that neuroE2 may be the primary mediator of certain types of cognitive responses, namely those relating to object recognition, but spatial learning and memory may depend on both neuro (direct production in the brain) and central (gonadal) E2 synthesis.

The collective data in rodents treated with AI have thus yielded conflicting findings as to whether this treatment affects cognitive responses. Potential conflicting data might be explained by differences in rodent strains used, sex, gonadectomized state, age at time exposure, specific AI administered, dose and duration of the treatment, and even which behavioral tests were used to assess learning and memory. Further work is also needed to determine whether developmental exposure of rodent models leads to long-term learning and memory deficits, as such treatment might disrupt organizational effects of T/E2 in the brain (27, 28, 107). The current mouse and rat studies suggest that *Src* and secondarily, *Psd95* expression in the hippocampus might be vulnerable to AI treatment, and such effects could disrupt synaptic plasticity that is essential for cognitive traits. Presumably, there are other genes and brain regions involved in learning and memory that might be affected by AI, which should be the focus of future studies.

## Avian models

Although avian models have not been used for gene deletion studies, these species have been investigated in terms of responses to aromatase inhibition. E2 might be important in sensory processing in the auditory region of the songbird forebrain, termed the caudomedial nidopallium (NCM), as when birds hear conspecific song, a surge in local E2 occurs (108–112). Neurons in this region express high amounts of aromatase enzyme and E2 receptors (ESRs) (108–112). Treatment of adult male zebra finches (*T. guttata*) with the AI, FAD (intramuscular injection of 100 μg in 10 μl of 0.75% saline), for 8 days resulted in reduced memory for training songs relative to saline-treated controls; however, auditory processing for novel songs did not differ between the two groups (113). The findings suggest that aromatase activity is required for neuronal memory for vocalizations in the songbird NCM. Similarly, acute aromatase inhibition with Vorozole (30 mg/kg) disturbed auditory processing in several brain regions selectively in the left hemisphere in male European starlings (*Sturnus vulgaris*) with the effects more prominent in March than in December or May/June for reasons that are uncertain (111). Photostimulated and E2-treated male European starlings exhibited a greater response rate to learn and respond to conspecific male song segments than those treated with saline or FAD (8.4 mg/100 ml avian saline in a micro-osmotic pump with a release rate of 0.25 ml/h) (114).

Male canaries (*Serinus canaria*) injected intraperitoneally with FAD within 2–5 min after the lights were turned on showed reduced motivation to sing and song acoustic stereotypy, an indicator of consistency over song renditions within a day's period, but all song measures returned to normal by the next day, suggesting that locally produced neuroE2 might act in a rapid manner to induce these learned vocal behaviors (115). Treatment of adult female canaries with FAD and testosterone induced song motor development resulted in changes in the song profile, and such alterations were linked with suppression of E2-sensitive transcripts, e.g., brain derived nerve growth factor (*Bdnf*) in the higher vocal center (HVC), but without altering the size of this brain region (116).

Other avian studies have examined the effects of AI on initial developmental brain programming. Treatment of juvenile male zebra finches with FAD (resulted in smaller neurons within the song motor centers- robust nucleus of the archistriatum (RA) and HVC but these histopathological changes were not associated with altered song behavior (117). Another study suggested FAD-treatment of posthatching male zebra finches from days 1 to 30 was not associated with changes in soma size in both song motor centers (118). Female zebra finches treated as embryos on day 3 of incubation with FAD (20 μg/5 μl saline) had gonadal sex reversal with a left ovotestis and right testis, larger syrinx, but this treatment did not alter the volume and soma sizes in the song system nuclei (HVC and RA), which the investigators attributed to the fact that testicular androgens and E2 synthesis are insufficient to masculinize the song system in females, although this treatment had a small but significant effect in demasculinizing one aspect of the neural song system (118, 119). While there are seemingly conflicting data as to whether local aromatase is required for internalization (where songbirds learn their songs by imitating an internalized auditory song model or potential template acquired from a parental figure) and production of song, several factors, including species differences, timing of aromatase suppression (developmental or in adulthood), and dose of FAD tested, could account for the disparate results. It is also not clear if developmental or later exposure to AI alters brain regions associated with the song system. On the other hand, such auditory memory defects might be reversed with aromatization recovery during the breeding season. Conflicting data might also be attributed to species examined, dose and type of AI tested, timing of exposure, and specific brain region studied. Thus, across avian models, aromatase-mediated E2 production in the auditory processing region(s) (NCM) is critical in song vocalization. It would be of interest to determine in neonatal rodents, whether administration of AI compromises their potential ability to learn various vocalization responses, including ultrasonic vocalizations (USVs).

Aromatase suppression, especially directly within the brain, might result in other cognitive dysfunctions in birds. Adult male zebra finches were treated directly within the hippocampus with an AI, 1,4,6-androstatriene-3,17-dione (ATD), ATD +E2, or ATD + G protein-coupled E2 receptor (GPER) agonist G1, and spatial memory assessed 72 hrs after the surgeries (120). Birds treated with ATD alone displayed compromised spatial memory, as evidenced by increased latency to reach a baited cup and made several mistakes in the process. However, co-treatment with E2 improved spatial memory as this group demonstrated the lowest retention latencies and made fewer mistakes. Those exposed to ATD and G1 resembled control animals. The post-synaptic protein (PSD95, a membrane-associated guanylate kinase important for synaptic strength) was lowest in the hippocampus of ATD-treated birds. Taken together, spatial memory in zebra finches is dependent upon local brain aromatase or neuroE2 and may act via GPERs. Conversely, treatment of non-breeding male and female Western scrub-jays (*Aphelocoma californica*), a food-caching corvid possessing excellent spatial memory skills, with FAD and E2 implants suggest that E2 treatment suppressed spatial memory, including searching efficiency and latency to retrieve the first item (121). Another study reported that FAD-treatment of zebra finches impaired retrieval of spatial memory, whereas, spatial memory acquisition was enhanced when E2 production was blocked (122). The opposing results may be due to testing of breeding vs. non-breeding birds, along with species differences, timing of exposure, test used to measure spatial learning and memory, and/or sex differences, albeit these were not specifically examined for in the current studies.

## Human aromatase studies

Aromatase activity affects brain and cognitive functions, not only across animal species, but in humans as well. This makes the animal studies cited above critical in lending insight into how aromatase activity and inhibition play a role in human cognition and behavior and diseases associated with such impairments. In the human brain, aromatase is particularly high in temporal and frontal brain areas; these regions are generally associated with learning, memory, and sensory processing (temporal lobes) and dopaminergic activity (frontal lobes). Interestingly, a 2006 study from the American Journal of Medicine identified relationships between sex steroid hormone gene polymorphisms and cognitive function in women and found important relationships that strongly implicate single nucleotide polymorphisms (SNPs) in genes associated with female sex steroids and cognition. Among them, subjects with a SNP in the aromatase gene (i.e., *CYP19* rs936306 CC genotype) scored significantly lower on a memory test of repeated recall; on the other hand, other SNPs in this gene (e.g., rs72824 GG genotype) have been associated with higher memory scores (123). The avian studies detailed above suggest that CYP19 expression in the NCM (43, 74), a brain region associated with auditory discrimination and song recognition memory (124, 125), may regulate such processes. Similarly, *CYP19A1* has been proposed to be a candidate gene regulating human cognitive functions associated with reading, speech, and language (126). This notion has been proposed based on the fact that examination of a Finnish, German, and several United States dyslexia cohorts reveals *CYP19A1* expression in the human brain is positively correlated with dyslexia susceptibility genes, including dyslexia susceptibility 1 candidate 1 (*DYX1C1)* and roundabout homolog 1 (*ROBO1)* (126). Variations in *CYP19A1* are also associated with dyslexia as a categorical trait and quantitative disruptions in reading, vocabulary, phonological processing, and oral motor skills (126). This same study also showed that ArKO mice have an increase in vertical neuronal density and occasional cortical heterotopia as observed in *Robo1* mutant mice and human dyslexia brains, respectively.

Another study investigated associations between *CYP19* gene polymorphisms with Alzheimer's disease (AD) (127). Although the vast majority of AI therapy is prescribed to treat breast cancer, this class of drugs can be used to treat other human clinical conditions. It has been prescribed to treat short stature in boys (128). Other conditions treated with AI include: infertility in women (129) and men (129), endometriosis (130), intravenous leiomyomatosis (131), and Klinefelter syndrome (132). Although one study tested the cognitive effects of AI in boys treated for short stature and found no relationship (133), more research is necessary to determine the long term effects of such therapies on cognitive function in both sexes and especially when administered at an early age.

Breast cancer is the most common cancer among women in the United States, and obesity, defined by excess adipose tissue, is a major risk factor (134). Since aromatase is highly expressed in adipose tissue, obese women experience greater E2 exposure than lean women, which renders them more susceptible to E2-induced tumors (135). Moreover, obesity associates with adipose tissue inflammation, which stimulates aromatase activity; a topic that has been extensively reviewed (136). Even lean women who display increased breast adipose tissue inflammation present with greater aromatase activity and increased risk of breast cancer (135). Thus, AI therapy represents a cornerstone of breast cancer treatment. This is also because two-thirds of breast cancer cases are ER+ tumors that depend upon E2 for growth and survival. High expression of aromatase in mammary adipose tissue makes it the major contributor to local E2 exposure. Thus, AI are often prescribed to women diagnosed with breast cancer. Notably, obese women have been shown to be somewhat resistant to the effects of AI (137), which may result in these women being prescribed higher doses and thereby experiencing even more severe side effects.

Several negative cognitive side effects, such as difficulty concentrating and forgetfulness, are commonly reported among women in AI therapies (138). The main cognitive impairments associated with AI therapy are verbal episodic memory and executive function impairments (139). The mechanism by which AI induce cognitive deficits presumably involves suppression of E2 signaling, with AI therapy in postmenopausal women resulting in undetectable blood concentrations of E2 (140). Tamoxifen (TAM), a competitive inhibitor of ER, has also been linked with cognitive dysfunction when used to treat post-menopausal breast cancer (138), suggesting that memory deficits associated with AI may involve suppression of ER signaling. Indeed, the vast majority of studies that have compared the cognitive effects of AI and tamoxifen have concluded that they result in analogous cognitive disruptions (27, 139, 141–143), although one study reported enhanced disturbances with TAM (142). Another study showed no cognitive alterations with AI therapy, but that study also found no changes in circulating E2 (144). Unlike TAM therapy (143), AI are also associated with musculoskeletal problems, including joint pain and arthralgia. However, both treatments are associated with bone health impairments (145). AI therapy among breast cancer patients was most likely to cause forgetfulness, followed by difficulty concentrating, hair loss, and numbness/tingling of extremities (138). A meta-analyses of 911 breast cancer patients treated with various AI treatments or TAM compared to control women further revealed that those treated with AI or TAM showed similar verbal learning and memory impairments (146). Treatment of breast cancer patients with the AI, An, has also been associated with memory deficiencies, especially in verbal memory, mood disturbances, somnolence, anxiety, fatigue, and hot flashes in some studies (14, 147–149).

A few reports though have found no cognitive changes in women receiving long-term AI treatment. One study reporting no cognitive disruptions with women receiving transdermal T + AI therapy (Letro 2.5 mg/day for at least 8 weeks) or placebo tablet also found no changes in circulating E2 (144). Similarly, an analysis with 111 women receiving An (1 mg/day for 5 years) compared to 116 women receiving a placebo tablet revealed no effects of AI on mental function (150). Notwithstanding, the majority of studies suggest that there is cause for concern that women receiving AI treatment might demonstrate later learning and memory deficits. The few reports above that found null effect with AI therapy might be attributed to the measures used to examine cognitive ability, population of women examined (in that due to genetics or other factors some women might be less susceptible to AI-induced cognitive dysfunction), dose and type of AI administered, and various other potential confounders. If most women who receive AI show later neural effects, the question is what, if anything, can be done to mitigate this risk. One possibility that has received recent attention is to encourage physical activity in these patients (151, 152).

## Potential remediation strategies for aromatase inhibition-related cognitive impairments

An interesting observation is that many women taking AI report significantly less physical activity (153). The reduced motivation to engage in physical activity might be attributed to musculoskeletal problems (e.g., joint pain, arthralgia) following AI therapy; physical inactivity may also contribute to and/or exacerbate those musculoskeletal problems. Indeed, there is a relationship between women reporting adverse musculoskeletal symptoms and reduced physical activity (153). Associations between E2 synthesis and increased pain responses or nocioception have also been reported in rodents (154–157). AI-induced pain in mice can be blunted by treatment with an antagonist for the transient receptor potential Ankyrin 1 (TRPA1) channel (158). Moreover, this study showed that such pain responses are absent in *Trpa1*^−/−^ mice (158). OVX rats administered a single dose of 1 or 5 mg/kg Letro had decreased mechanical withdrawal thresholds without changing thermal sensitivity, suggestive that central aromatase might guide the former response (159). Male rats repeatedly injected with 5 mg/kg Letro had mechanical, but not thermal, hypersensitivity that ceased when drug dosing was discontinued. A single dose of 5 mg/kg Letro or Exemestane to male rats enhanced flinching behavior induced by intraplantar injection of 1,000 nmol of ATP. Within the dorsal root ganglia, both small and medium sized sensory neurons from Letro-treated rats were more excitable with increased action potential firing and lower resting membrane potential, which may augment nocioceptive behaviors in AI treated rats (159).

There is evidence in rodents that neuroE2, signaling through ESR1, increases physical activity (160). Moreover, estrous cycling in rodents robustly correlates with physical activity, with the peek estrus period associating with greatest physical activity (161). In fact, monitoring of female rodent physical activity is an accurate predictor of where they are in the estrous cycle. Interestingly, in humans, the suppressed willingness to engage in physical activity after AI therapy is not solely explained by musculoskeletal abnormalities (153), implying that loss of central E2 signaling may also explain part of the suppression of time spent exercising. Notably, the effect of aromatase inhibition on physical activity may be sex-specific since preclinical studies have shown that physical activity is not affected in male mice with aromatase suppression; however, this study did not assess female mice (162). Another experiment with rodents discovered that genetic ablation of aromatase resulted in increased adiposity in both males and females. They went on to show, in the females, that the increase in weight gain was due to reduced physical activity; however, they did not assess physical activity in the males (163). It may be the case that aromatase-mediated E2 increases physical activity in females whereas other factors (e.g., T) are more important regulators of physical activity in males. Human studies have also shown reduced physical activity specifically among women on AI (164). This finding likely has important clinical ramifications, as physical inactivity exacerbates musculoskeletal dysfunction, as mentioned above. Moreover, physical inactivity associates with poor cognition, whereas increasing physical activity improves learning and memory ability, as extensively reviewed (165). Mechanistically, physical activity enhances neurogenesis and synaptogenesis in the hippocampus, a region of the brain governing cognitive function in humans and rodents (165–168).

E2 enhances neurogenesis and synaptogenesis (19, 169, 170), implying that E2 derived from aromatization of T in the brain of males may also have neuroprotective effects (171, 172). In contrast, AI treatment reduces hippocampal neurogenesis and synaptogenesis (173, 174). In isolated rat hippocampal neurons, Letro treatment reduces E2 synthesis with an accompanying decrease in density of spine synapses, presynaptic boutons, and decreased protein expression of the spine-marker, spinophilin (PPP1R9B) and the pre-synaptic marker, synaptophysin (SYP) (173, 174). Moreover, culture of hippocampal neurons reveals that Letro phosphorylates aromatase and reduces estradiol synthesis, whereas, treatment of cells with E2 results in phosphorylation of the enzyme and increased aromatase protein expression, suggesting estradiol synthesis in hippocampal neurons is subject to autocrine regulation (175).

It is thus reasonable to postulate that enhanced physical activity may be a critical adjuvant to AI therapy in humans, at least among women. Not only might it offset the musculoskeletal symptoms that occur commonly with use of AI, but it may also mitigate against the cognitive side effects of these drugs. Select animal data suggest that AI may adversely affect nocioceptive behaviors (158, 159). Similar findings have been reported in women treated with AI (176–178). One study assessing the effects of exercise to offset such symptoms associated with AI showed that exercise reduced pain sensitivity among women taking this therapy (179). It is unclear whether the CNS-specific changes with AI therapy may mediate the subjective musculoskeletal, cognitive, and nocioceptive responses in humans or animals, but both AI and exercise have been shown to directly induce brain changes. In humans, positron emission tomography (PET) scanning revealed significant impairments in metabolic activity in the medial temporal lobes in a group of women not reporting subjective symptoms of cognitive impairment; this may suggest that brain changes precede the detection of cognitive deficits and warrant treatments to prevent such brain changes from taking place while on AI therapy (13). Exercise may indeed be a low-cost and promising therapy to mitigate AI-induced cognitive disruptions and other neurobehavioral disorders (151, 152). A recent control trial study, termed Exercise Program in Cancer and Cognition (EPICC), has in fact been initiated to test this notion in that it will measure the effects of aerobic exercise on cognitive function in postmenopausal women with breast cancer and who are being treated with AI therapy (180).

## Aromatase and alzheimer's disease

This section will consider whether declining aromatase activity, especially within the brain, or genetic mutation/ablation of *CYP19*/*Cyp19* in humans and rodents, respectively, is associated with an increased risk for AD. Major findings of these studies are summarized in Tables 1, 2. One human study investigated associations between *CYP19* gene polymorphisms with Alzheimer's disease (AD) (127). Those authors studied nine polymorphisms in 207 AD patients, 23 cases of mild cognitive impairment, and 233 controls. These were all participants of the OPTIMA (196) study. The most salient finding from that study was that significant associations were found between variants of the aromatase gene and risk of AD specifically in women. Relatively similar results were reported in another cohort study representing 1,686 women participating as part of the Washington Heights Inwood Columbia Aging Project with six *CYP19* single nucleotide polymorphisms (SNPs) present in women of predominantly Caucasian ancestry that are associated with increased risk of AD and 2 SNPs in this gene present in women of mixed and Hispanic origin linked are linked with decreased risk of AD (181). Several other studies have linked *CYP19* genetic variants and interaction of this gene with other ones, including butyrylcholinesterase (*BCHE*), *CYP17*, and interleukin 10 (*IL-10*) and susceptibility to developing AD (182–189). Taken together, these human studies support that an important relationship exists, in particular in females, between brain aromatase activity and normal cognitive function with genetic disruptions in this gene being associated with AD in older women. Depending on the location of the SNPs within the *CYP19* gene, it might affect aromatase activity, substrate binding, and/or result in disequilibrium in this enzymatic pathway.

**Table 1:** Human studies linking *CYP19* gene polymorphisms and Alzheimer's disease (AD)^*^.

**References**	**Study population and design**	**Major findings**
(127)	Nine polymorphisms in in the *CYP19* gene were examined in 207 cases of AD, 23 cases of mild cognitive impairments (MCI), and 233 controls from the OPTIMA cohort that included men and women.	Consistent findings were observed between AD and MCI cases. Significant interactions were identified with select polymorphisms and sex. Importantly, all associations with Cyp19 polymorphisms and AD were identified entirely in women.
(181)	SNPs in the *CYP19* gene were examined in the 1686 women who participated in the Washington Heights Inwood Columbia Aging Project and correlated with their risk for AD.	6 SNPs associated with risk for ASD in Caucasian ancestry women. 2 SNPs were associated with decreased risk for this disorder in women of admixed/Hispanic ancestry. 2 SNPs were protective in women of predominantly African-based ancestry.
(182)	Nine polymorphisms in in the *CYP19* gene were examined in 394 AD patients and were compared to 469 control subjects, and haplotypes were identified using single-locus and haplotype approaches.	3 adjacent SNPs differed between AD and control groups. Both haplotype approaches identified an ~60% increase in the risk for AD for one haploytype. Genetic variation in brain *CYP19* may increase the risk for AD.
(183)	Polymorphisms in the 5′-UTR of CYP19 and a non-K/K variant in the butyrilcholinesterase *(BCHE*) enzyme were examined in 187 sporadic AD patients and 172 control subjects in a Spanish population.	The *CYP19* C/C genotype was increased in AD patients also carrying the BCHE non-K allele relative to controls. Polymorphisms in *CYP19* and *BCHE* may interact to affect risk for AD.
(184)	18 SNPs spanning the 5′ UTR region and the entire coding region of *CYP19* was examined in 227 patients with AD and 131 control spouses.	A haplotype in block 1 and haplotype in block 2 increased the risk for developing the disease by 2-fold in apolipoprotein E *_ε_4* (*APOE _ε_4)* carriers.
(185)	Potential interactive effects of SNPs in *CYP19* (5' UTR GG) and *IL-10* (-1082, GG) genotypes were examined in 231 AD patients and 194 healthy controls.	Subjects with the *CYP19* (GG) and *IL-10* (GG) genotypes showed a 6-fold risk reduction in developing AD compared to individuals without these genotypes.
(186)	3 SNPs (res12907866, rs17601241, and rs4646) in *CYP19* were examined in 319 AD patients and 110 controls.	While no linkage was fond in *CYP19* SNPs and AD risk, genetic variation in this gene was associated with earlier onset of AD age development in women, specifically the rs4636 genotypes carrying a T allele. This association was independent of a similar correlation found with the *APOE _ε_4* allele.
(187)	SNPs in *CYP17* and *CYP19* were examined in 235 women with Down syndrome (DS), who ranged in age from 31 to 67 years and did not show any signs of dementia at the initial examination.	4 SNPs in *CYP19* were correlated with 2-fold increase risk for AD with 3 only significant individuals without an *APOE _ε_4* allele. 4 SNPs in *CYP17* were linked with a 2.5-fold increased risk for AD, which was independent of APOE genotype. Individuals carrying high risk alleles in both *CYP17* and *CYP19* were associated with ~4-fold elevated risk for AD and increased sex hormone binding globulin in post-menopausal women. Variants in both genes involved in E2 bioavailability was associated with decreased age of AD onset in women with DS.
(188)	SNPs in *CYP19* and *IL-10* were examined in 1757 AD patients and 6294 controls enrolled as part of the Epistasis Project.	Women with SNP rs1065778 GG in the *CYP19* gene was associated with increased odds ratio risk of developing AD. An interaction existed in women between *IL-10* rs1800896 and *CYP19A1* rs1062033 and increased risk for AD. Findings suggest that decreased serum E2 and neuroE2 may increase neuro-inflammation and risk for AD.
(189)	SNPs in *CYP19A1* were assessed in a Chinese Han population that included 207 patients with AD and 256 control individuals.	Within *APOE _ε_4* carriers, a different distribution of the G allele and the AG + GG genotype of *CYP19A1* rs3751592 were found in AD patients compared to control individuals. No differences were identified in the distribution of *CYP19A1* rs1065778 between AD patients and controls, independent of *APOE _ε_4* genetic status.

* *Meanings of abbreviations used in the Table are detailed in the manuscript*.

**Table 2:** Rodent Model Studies Linking *Cyp19*/CYP19 gene and protein expression and AD-like signs^*^.

**References**	**Animal model**	**Treatment/dosing regimen**	**Major findings**
(190)	5xFAD mouse model for AD	None	*Cyp19* mRNA and protein expression lower in the hippocampus in females but not males in 5xFAD mice.
(191)	Male and Female 3xTgAD mice	Some animals were gonadectomized and others were left intact. Some of the gonadectomized individuals were implanted subcutaneously with hormone treatment (HT) over 90 days and were replaced at 6 months of age, and consisted of E2 (0.25 mg) and progesterone (25 mg) or 12.5 mg T for females and males, respectively. Within these above groups, some were treated with An (0.4 mg/animal/day).	Those treated with An had An serum levels of 10.19 ng/mL and brain levels were detected at 4.7 pg/mL. An increased Aβ plagues but not APP/Aβ-immunoreactivity in the hippocampus of intact 3xTgAD females compared to controls. An increased number of Aβ- compared to APP/Aβ-positive hippocampal CA1 neurons in intact and OVX female mice. An decreased the APP/Aβ plaque load in 9 month old intact and OVX female 3xTgAD mice. Central and brain aromatase inhibition might differentially affect amyloid type proteins and might affect extraneuronal to intraneuronal ratio of accumulation.
(192)	Deletion of the FSR-receptor (FORKO) and two transgenes with one expressing the β-amyloid precursor protein Swedish mutation (APPsw) and the other expressing presenilin-1 lacking exon 9 (PS1Δ9) and wild-type (WT) mice	No treatment of transgenic mice. Primary hippocampal neurons and glia from 5-day-old WT mouse pups were cultured treated on day 5 with Letro (1 μM) or vehicle control for 24 h.	This mouse model has chronic E2 deficiency. the brains of these mice have marked hypertrophy of cortical neurons and astrocytes and increased number of activated microglial cells. Aβ plaques did not differ but such lesions appeared less compact and larger than respective control mice. Letro treatment of cortical neural cultures from control mice revealed similar glial abnormalities as identified in this AD mouse model.
(193)	ArKO mice were bred to APP23 transgenic mice to generate E2-deficient APP23 mice	None	These trangenic mice demonstrate reduced brain E2 concentrations, early-onset and increased brain production and deposition of Aβ plaques. Microglial culture from these mice show impaired ability to clear and degrade Aβ plaques. Such plaque abnormalities not found in OVX APP23 mice, suggesting brain aromatase deficiency/estrogen depletion as being an important determinant for developing AD-associated neuropathologies.
(194)	APP23 female mice with genetic deficiency of aromatase [APP/Ar(^+/−^)] OVX APP23 (APP/OVX) mice	3 months old, APP/OVX mice or APP/Ar^+/−^ female mice were implanted subcutaneously with a E2 pellet (1.7 mg or 18.9 μg/day), a 17α-estradiol pellet (1.7 mg or 18.9 μg/day), a genistein pellet (24 mg or 26 μg/day), a black cohosh pellet (24 mg or 26 μg/day), or placebo pellet	APP23 OVX mice contain estrogen in the brain. Only APP/Ar(+/-) but not APP23 OVX mice had reduced Aβ plaques following E2 or genistein (G) treatment. E2 and G treatment to APP/Ar(+/-) resulted decreased BACE1 mRNA and protein expression. E2 or genistein supplementation might reduce AD neuropathological changes by increased neuroE2 concentrations.
(195)	Male mice generated from ArKO combined with APP23	None	Male transgenic mice show reduced brain plague formation, improved cognitive functions, increased NEP activity, and reduced expression of BACE1. Findings suggest that in males an increase in endogenous T due to removal of the CYP19 enzyme protects against AD. This protection might be due to increase T downregulation of BACE1 activity leading to decreased β-amyloid production and upregulation of NEP to enhance β-amyloid degradation.

* *Meanings of abbreviations used in the Table are detailed in the manuscript*.

A handful of studies have examined aromatase expression in AD patients compared to controls. In three studies, lower aromatase mRNA or protein expression was identified in neurons of the hypothalamus, hippocampal, and basal brain regions (127, 197, 198). One study, however, found no difference in aromatase activity in the human frontal and temporal cortex in AD patients (199). Luchetti et al. (200) reported that in AD patients *CYP19* mRNA expression was greater in the astrocytes within the prefrontal cortex region. Collectively, the studies suggest that aromatase expression, especially within the neurons of the hippocampal and basal region and glial cells, may serve as a reliable biomarker for AD patients.

Rodent models have also been useful in deciphering the roles of aromatase in AD, as shown in Table 2. Studies showing that *Cyp19* mRNA and protein expression are lower in the hippocampus in females but not males in a mouse model for AD (5xFAD) provide further evidence that normal expression of this enzyme in the brain is important in conferring against cognitive impairments and that females might be more vulnerable than males (190). To examine further the role of central and brain aromatase in AD mouse models, male and female were either gonadectomized or left intact, some of the gonadectomized individuals were treated with replacement hormone therapy, and other individuals were treated with An (additional details provided in Table 1) (191). Those treated with An had An serum levels of 10.19 ng/mL and brain levels were detected at 4.7 pg/mL. An increase in Aβ plagues but not APP/Aβ-immunoreactivity was detected in the hippocampus of intact 3xTgAD females compared to controls. An increased number of Aβ- compared to APP/Aβ-positive hippocampal CA1 neurons was identified in intact and OVX female mice, but this AI treatment decreased APP/Aβ plaque load was identified in 9 month old intact and OVX female 3xTgAD mice. The findings suggest that central and brain aromatase inhibition might differentially affect amyloid type proteins and might affect extraneuronal to intraneuronal ratio of accumulation.

Other mouse models for AD also suggest that aromatase/E2 synthesis confers protection against neuropathological lesions. One such chimeric mouse has chronic E2 deficiency due to complete deletion of the FSR-receptor knockout (FORKO) and two transgenes with one expressing the β-amyloid precursor protein Swedish mutation (APPsw) and the other expressing presenilin-1 lacking exon 9 (PS1Δ9), and the brains of these mice have marked hypertrophy of cortical neurons and astrocytes and increased number of activated microglial cells (192). While number of Aβ plaques did not differ, such lesions appeared less compact and larger than respective WT mice. Letro treatment of cortical neural cultures from control mice revealed similar glial abnormalities in this AD mouse model, further emphasizing the importance of aromatase and E2 synthesis in preventing such neural abnormalities associated with AD (192). To elucidate the role of aromatase/E2 synthesis in AD, ArKO mice were paired with APP23 transgenic mice (another mouse model for AD) to generate E2-deficient APP23 mice (193). Such mice demonstrate reduced brain E2 concentrations, as well as early-onset and increased brain production and deposition of Aβ plaques. Microglial culture from E2-deficient APP23 mice show impaired ability to clear and degrade Aβ plaques. Such plaque abnormalities were not found in OVX APP23 mice, implicating brain aromatase deficiency/E2 depletion as being an important determinant for developing AD-associated neuropathologies. This study also showed postmortem analyses of brains from women with AD compared to those without this disease had lower levels of total and free E2, which was also reduced in the circulation. To determine whether E2 treatment might affect AD neuropathological changes in APP23 female mice with and without aromatase deficiency (APP/Ar(+/−)) and those lacking central E2 due to gonadetcomy (APP/OVX), both groups were provided estrogenic chemicals, e.g., ethinyl E2 or genistein (Details in Table 1) (194) These studies revealed that APP23 OVX mice contain E2 in the brain. Only APP/Ar(+/−) but not APP23 OVX mice had reduced Aβ plaques following E2 or genistein (G) treatment. E2 and G treatment to APP/Ar(+/−) resulted decreased beta-secretase (BACE1) mRNA and protein expression. Results suggest that E2 or genistein supplementation might reduce AD neuropathological changes by increasing E2 concentrations in the brain.

A follow-up study by this same group revealed male mice generated from this same above cross (ArKO combined with APP23) have reduced brain plague formation, show improved cognitive functions, increased NEP activity (neprilysin, which mediates Aβ clearance), and reduced expression of BACE1, suggesting in males an increase in endogenous T due to removal of the CYP19 enzyme protects against AD (195). This protection might be due to T-induced downregulation of BACE1 activity leading to decreased β-amyloid production and upregulation of NEP to enhance β-amyloid degradation (195).

## Conclusions and future directions

With the escalating usage of AI therapy to treat breast cancer and other clinical conditions, it is imperative to understand the potential effects such drugs can exert on the CNS. Aromatase conversion of T to E2 in the brain is integral at varying points in the lifespan. Early in development, this enzyme can help guide organizational programming of the brain (27, 28, 107). Later in life, aromatase is needed for full manifestation of sexual behaviors in males, such as song production (111, 113–115). However, animal and human studies reveal that aromatase is also needed to support normal cognitive function and other behaviors in females (2, 19–22). Although the mechanisms are yet to be elucidated fully and may be multifactorial, it is clear that AI treatment can result in cognitive dysfunction in animals, as well as humans.

The neurobehavioral disturbances induced by AI likely depend upon when during the lifespan such drugs are administered. If during the perinatal period or the onset of sexual maturity, it could assumingly disrupt normal organizational-activational programming, as shown in various animal models (201, 202). Post-menopausal women and older animals treated with such drugs are more likely to show memory deficits, increased pain response, and other emotional responses (10–18).

Avian and rodent models treated with AI have been useful in understanding and predicting the potential effects in patients treated with AI. However, effects can vary depending on the species tested, AI employed, and dose. In this aspect, ArKO mice that are systemically devoid of the aromatase enzyme might be a preferred animal model in attempting to understand the clinical sequelae of full suppression of aromatase. One potential limitation though with the current ArKO mouse models is that they lack the aromatase enzyme throughout their lifetime (29–32). To pinpoint how ablation of aromatase at specific windows of time affects various neurobehavioral domains, a conditional knockout, especially one lacking this enzyme in different brain regions, is essential. Alternatively, adenovirus or other targeted approaches to delete aromatase expression in specific brain areas, such as the hippocampus and prefrontal cortex, that are required for long-term memory consolidation, can be employed. Such an adeno-associated viral vector approach has been used to silence *Esr1* in the ventromedial (VMN) nuclei of the hypothalamus (203). A similar approach has been used to suppress *Esr2* in the MPOA region from the prepubertal through adult period in male mice, which resulted in decreased aggressive but not sexual behaviors at adulthood (204). In contrast, pre-pubertal through adult suppression of *Esr2* in the MeA disrupted male preference for receptive females compared to non-receptive females. A follow-up study by this group revealed that knocking down *Esr1* in the MeA or MPOA in intact male mice at 21 days of age resulted in reduced sexual and aggressive behaviors when administered into the MeA but only decreased sexual but not aggressive behaviors with MPOA treatment (205). Pre-pubertal suppression of MeA also resulted in reduced neurons within this brain region.

In rodents, the effects of AI might also depend upon sex, AI tested, and gonadal state of the individual. For instance, treatment of intact male rats with Letro seems to improve spatial working memory (99, 100). However, mixed results have been reported in females, which likely depend upon the AI tested, whether the animals are intact or OVX, and type of cognitive test (101–103).

**Figure 2:**
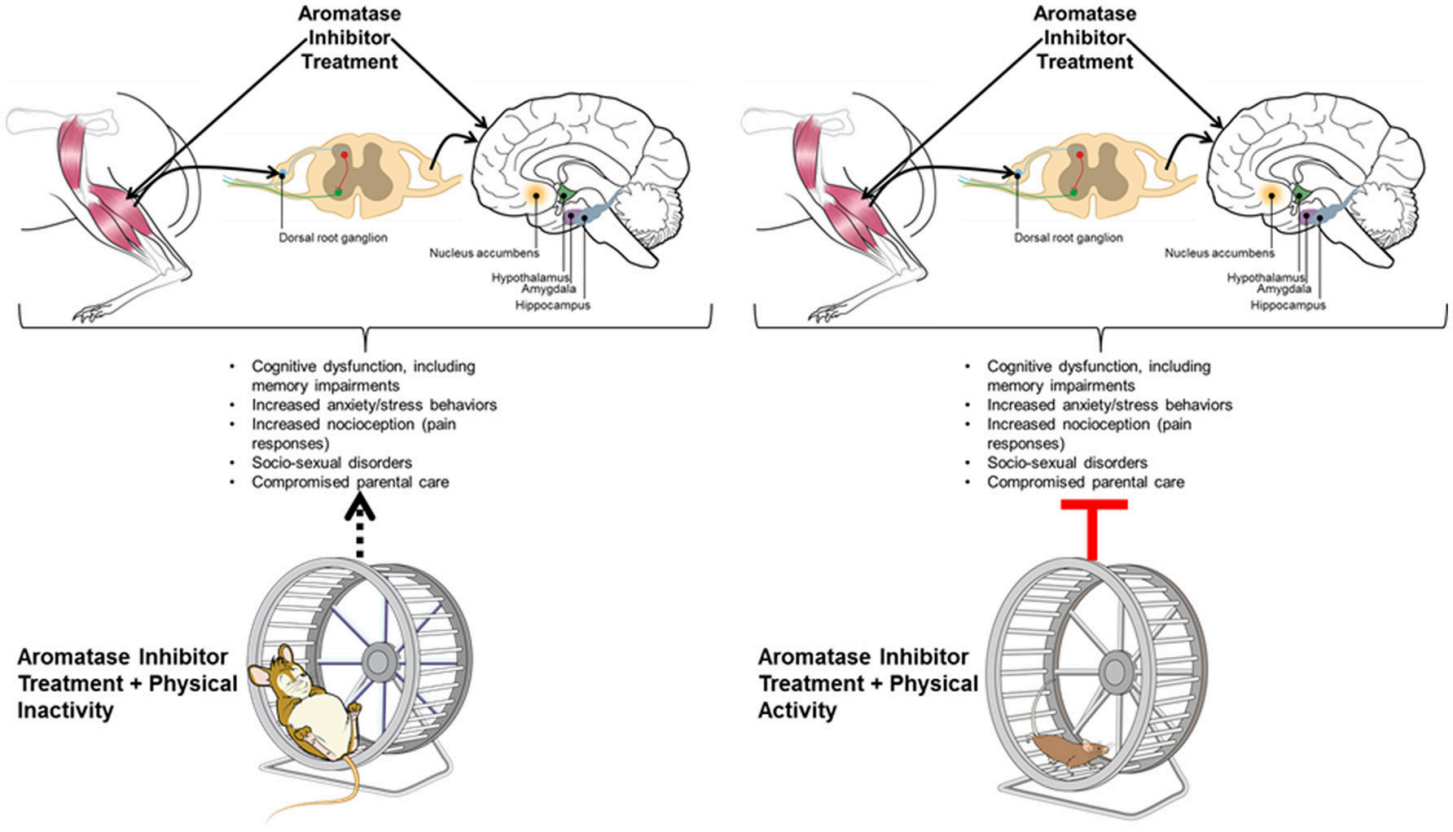
Hypothetical model representing relationships between central and peripheral mechanisms leading to cognitive impairments associated with aromatase inhibition (AI) therapy, and suggesting physical activity as a potential remediation strategy for prevention of such effects. As shown on the reader's left-hand side of the figure, AI therapy is known to impair cognitive function, induce musculoskeletal symptoms, and is associated with suppressed voluntary physical activity in humans and animals. This constellation of symptoms may be related centrally, whereby AI adversely affects key brain regions (e.g., nucleus accumbens, as discussed in the Conclusions, this is the primary brain region associated with motivation for physical activity; hypothalamus and amygdala- as discussed in the Conclusions, these brain regions are linked with central metabolic and emotive control; hippocampus, associated with a variety of cognitive functions including learning and memory) which may lead to physical inactivity and contribute to or exacerbate AI-associated musculoskeletal effects. Similarly, AI-associated musculoskeletal effects, i.e., increased pain or nocioceptive responses, may further suppress motivation to engage in physical activity. As shown on the reader's right-hand side, increasing physical exercise, however, may enhance neurogenesis within all four of these brain regions (nucleus accumbens, hypothalamus, amygdala, and hippocampus), improve overall cognitive function, and lessen musculoskeletal/pain symptoms, thereby improving the main adverse symptoms associated with AI.

Based on the majority of investigations reporting cognitive deficits in women receiving AI therapy, a big and open-ended question is whether these women are at greater risk for developing AD (134, 136). Declining E2 levels at menopause renders women more susceptible to cognitive deficits and AD, suggesting E2 replacement therapy might be beneficial in this aspect (206). Aromatase (*CYP19*) gene SNPs have been associated with increased or, for a few SNPs, decreased risk for AD (127, 181). In general, reductions in aromatase expression or activity are associated with AD, especially in women (127, 197, 198). A further protective role of brain aromatase is suggested by the fact that aromatase activity is elevated in astrocytes following brain injury (207). *CYP19* mRNA expression is also elevated in astrocytes of the prefrontal cortex region in some AD patients (200). Rodent models for AD and those where such models have been combined with ones lacking *Cyp19* gene provide robust support that normal expression of aromatase, especially within the neurons, is associated with less risk for AD-like cognitive impairments (190, 192, 193), and that females might be more vulnerable than males (190). Investigatory studies examining whether women who received AI are more likely to develop AD are thus needed. If such is the case, it is important to consider strategies, such as physical activity, that can be implemented early on to reduce this risk. Given the importance of physical activity in combating many of the AI-linked neurobehavioral disruptions (151, 179), physicians should be actively encouraging women on AI therapy to engage in routine physical activity. Herein, we cite evidence pointing to a unified mechanism potentially linking physical activity to protection against cognitive and metabolic impairments often associated with AI therapy. Figure 2 summarizes mechanisms linking central and peripheral consequences of AI therapy. Future human, such as the recently initiated EPICC study (180), and rodent model studies are essential in understanding whether physical activity can mitigate AI-induced CNS disorders and thereby improve memory impairments and other clinical signs in women receiving such therapies.

From being diagnosed to treated, women with breast cancer have already endured an enormous emotional toll. It is thus essential that we understand why such women who receive AI are more likely to experience memory deficits, increased pain responses, and other CNS and musculoskeletal affects. This will allow for the development of strategies to alleviate such negative side effects, thus allowing women to recover fully from both a physical and mental health standpoint. Exercise, both during the time of receiving such drugs and after, appears to hold the key in maintaining normal cognitive function in these women and the potential beneficial mechanisms of physical activity should be tested in animal models treated with AI or transgenic mice lacking aromatase enzyme at certain time points and/or brain regions.

## Author contributions

CR, DS, and VV-P researched this area and drafted and revised the manuscript.

### Conflict of interest statement

The authors declare that the research was conducted in the absence of any commercial or financial relationships that could be construed as a potential conflict of interest.
